# Sodium selenite prevents suppression of mucosal humoral response by AFB_1_ in broiler’s cecal tonsil

**DOI:** 10.18632/oncotarget.17105

**Published:** 2017-04-13

**Authors:** Chunyu Liu, Zhicai Zuo, Panpan Zhu, Zhixiang Zheng, Xi Peng, Jing Fang, Hengmin Cui, Yi Zhou, Ping Ouyang, Yi Geng, Junliang Deng, Yu Sun

**Affiliations:** ^1^ Key Laboratory of Animal Diseases and Environmental Hazards of Sichuan Province, College of Veterinary Medicine, Sichuan Agricultural University, Chengdu, Sichuan, PR China; ^2^ College of Veterinary Medicine, Sichuan Agricultural University, Chengdu, Sichuan, PR China; ^3^ College of Life Sciences, China West Normal University, Nanchong, Sichuan, PR China; ^4^ Life Science Department, Sichuan Agricultural University, Yaan, Sichuan, PR China

**Keywords:** aflatoxin B_1_, selenium, IgA+ cell, immunoglobulin, cecal tonsil

## Abstract

Aflatoxin B_1_ (AFB_1_), the most common mycotoxin in human food and animal feed, produces hepatotoxic, genotoxic and immunosuppressive effects in multiple species. Selenium (Se) has emerged as an important element in the dietary prevention of various toxic agents. The present study was designed to scrutinize the protective effects of sodium selenite on the histological lesions and suppression of mucosal humoral response in the cecal tonsil generated by AFB_1_. A total of 156 one-day-old broilers were divided into four groups and fed on basal diet (control group), 0.6 mg/kg AFB_1_ (AFB_1_ group), 0.4 mg/kg Se supplement (+Se group), and 0.6 mg/kg AFB_1_ + 0.4 mg/kg Se supplement (AFB_1_+Se group) respectively for 21 days. Our results showed that 0.4 mg/kg Se supplement in broiler's diets could improve the AFB_1_-induced histological lesions in the cecal tonsils including the depletion of lymphocytes in the lymphatic nodules as well as the shedding of microvilli in the absorptive cells. Moreover, Se could restore the decreased number of IgA+ cells and expression levels of pIgR, IgA, IgG, and IgM mRNA induced by AFB_1_ to be close to those in the control group. These results demonstrated that 0.4 mg/kg supplemented dietary Se in the form of sodium selenite could protect the cecal tonsils from the histological lesions and suppression of the mucosal humoral response provoked by 0.6 mg/kg AFB_1_. Our study may provide new experimental evidences for better understanding of AFB_1_-induced damage of mucosal immunity and protective effect of Se against this toxin.

## INTRODUCTION

Aflatoxins are mainly produced by *Aspergillus flavus* and *A. parasiticus* and found in various agricultural commodities and many food products [1]. Among the aflatoxins identified, aflatoxin B_1_ (AFB_1_) is the most abundant toxic metabolite and a well-known global carcinogen, causing mutagenic, carcinogenic, teratogenic and immunosuppressive effects on human and multiple animal species [2]. AFB_1_ may interfere with the normal process of protein synthesis as well as inhibition of several metabolic systems thus causing damages to various organs [3, 4]. With respect to human, the International Agency for Research on Cancer (IARC) classifies AFB_1_ within class 1 of human carcinogens [5].

The immunotoxic effects induced by AFB_1_ have been well documented in the literature, including innate immunity, cell-mediated and humoral response [1, 6, 7]. AFB_1_ has been shown to inhibit the development of thymus and bursa of Fabricius [8, 9], to depress the mitosis of B cells [10], to reduce the weight of lymphoid organs [11], to suppress antibody production [12–14] and to decrease the population and phagocytic capacity of macrophages [15].

Selenium (Se) is essential for the efficient and effective operation of many aspects of the immune system in both animals and humans [16]. Se supplementation has been found to regulate the function of B cells, T cells, neutrophils and NK cells [17]. Moreover, supplementation of Se in the form of sodium selenite was reported to have some protective action against the toxic effects of aflatoxin [11]. Early studies have revealed that Se could modify the disease process [18], and counteract the AFB_1_-induced adverse effects such as the retarded development and histopathological lesions of immune organs, reduced percentages of T cell subsets, along with decreased contents of antibodies [11, 13, 19, 20].

Cecal tonsils were the most immunologically mature lymphoid organ, being in the proximal one third of the paired tubular cecum, which laid along each side of the large intestine. More diffuse lymphoid tissue and unorganized lymphatic nodules were present both in the mucosa and submucosa [21]. Thus, cecal tonsils were the largest lymphoid organ of the gut-associated lymphoid tissue in avian [22] and played a crucial role in mucosal immunity. It has also been shown that cecal tonsils contain antibody-producing cells that direct humoral immune responses [23]. However, there were no systematic reports regarding the effect of AFB_1_ on the histology and mucosal humoral immunity of cecal tonsils along with the protective roles of Se against this toxin.

In the present research, experiments were conducted to examine the protective functions of dietary sodium selenite from the AFB_1_-induced histological lesions, and impaired mucosal humoral immunity of broiler's cecal tonsils by determining the histological structure, the number of immunoglobulin A-positive (IgA+) cell and expression levels of immunoglobulin mRNA. The outcomes from the present research could provide helpful insights for therapeutic measures to bring down the AFB_1_-induced suppression of mucosal immune response in humans and animals.

## RESULTS

### Histological changes in the cecal tonsil

**Figure 1:**
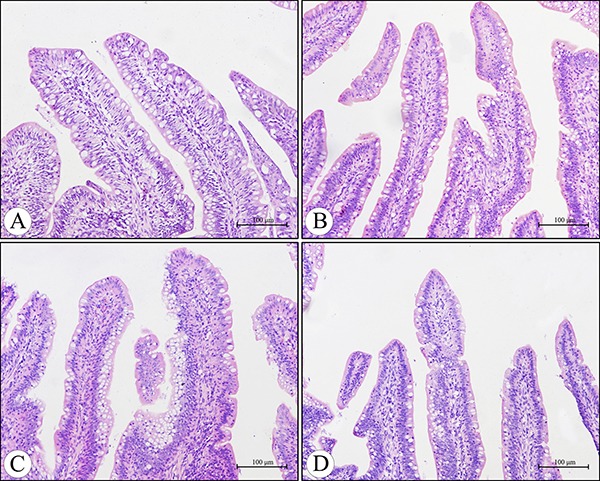
Histological structure of the villi in the cecal tonsil at 21 days of age under light microscopy (H.E staining) Compared with the control group (**A**), no lesions were observed in the villi of the cecal tonsils in the AFB_1_ group (**B**), AFB_1_ +Se group (**C**) and +Se group (**D**). Bars = 100 μm.

**Figure 2:**
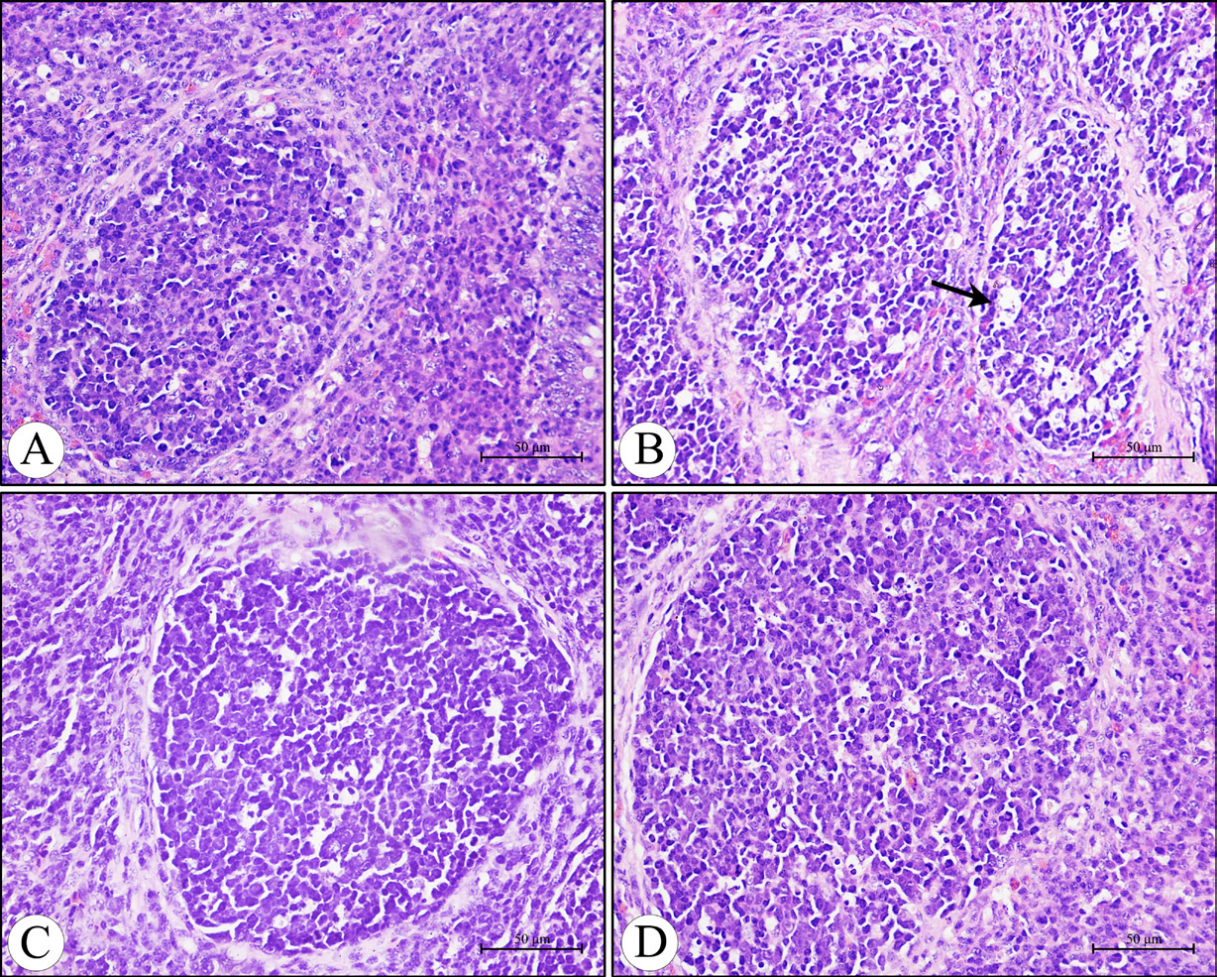
Histological structure of the diffuse lymphoid tissue and lymphatic nodules in the lamina propria of the cecal tonsil at 21 days of age under light microscopy (H.E staining) (**A**) The control group. (**B**) The AFB_1_ group showing the depletion of lymphocytes and presence of many vacuoles (arrow) in the lymphatic nodules. (**C**) The AFB_1_+Se group. (**D**) The +Se group. Bars = 50 μm.

**Figure 3:**
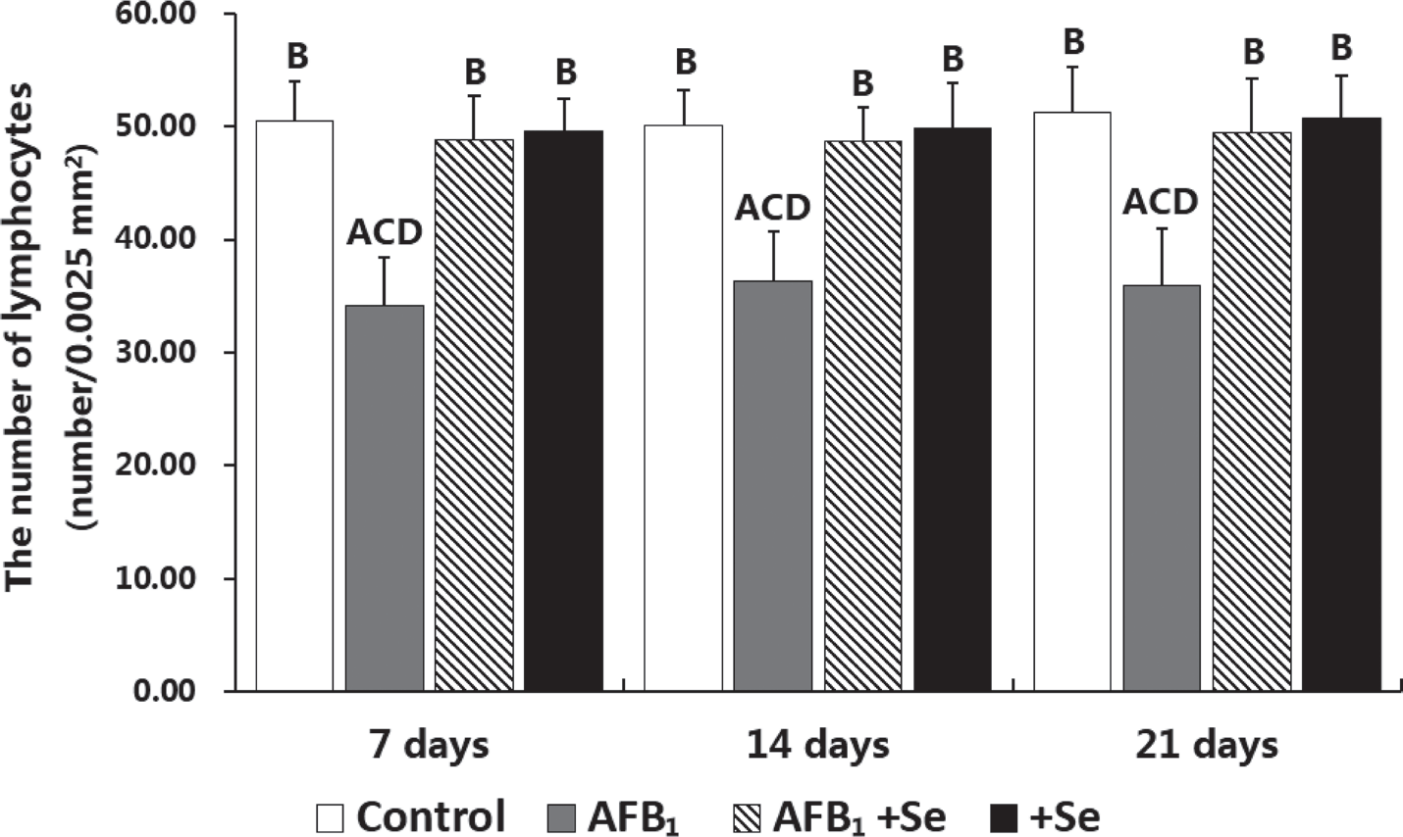
The number of lymphocytes in the lymphatic nodules in the cecal tonsil Note: Data are presented with the means ± standard deviation (*n* = 6). Letters A, B, C and D represent the significant difference (*p* < 0.01) between the group and control group, AFB_1_ group, AFB_1_+Se group, and +Se group, respectively. Letters a, b, c and d represent difference (*p* < 0.05) between the group and control group, AFB_1_ group, AFB_1_+Se group, and +Se group, respectively.

Microscopically, compared to the control group, no lesions were observed in the villi of cecal tonsils in the AFB_1_ group, AFB_1_+Se group and +Se group (Figure 1). However, the depletion of lymphocytes and presence of many vacuoles were visible in the lymphatic nodules in the AFB_1_ group, and no histological changes were observed in these places in the other three groups (Figure 2). Furthermore, the microscopic quantitative analysis also revealed a significant decline in the number of lymphocytes in the lymphatic nodules in the AFB_1_ group (*p <* 0.01) at 7, 14 and 21 days of age compared with the other three groups, and no significant changes in these values occurred among the control group, AFB_1_+Se group and +Se group during the experiment (*p* > 0.05) (Figure 3).

**Figure 4:**
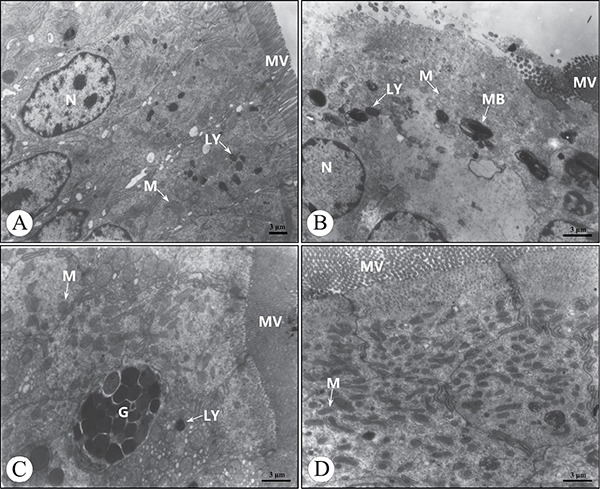
Ultrastructure of the absorptive cells in the cecal tonsil at 21 days of age under transmission electron microscopy (**A**) The control group. (**B**) The AFB_1_ group. (**C**) The AFB_1_+Se group. (**D**) The +Se group. Bars = 3 μm. MV, microvilli; N, nucleus; G, goblet cell; M, mitochondrion; LY, lysosome; MB, myeloid body.

Ultrastructurally, a few absorptive cells in the AFB_1_ group showed the shedding of microvilli, reduced number of mitochondria cristae, decreased cytoplasmic electron density, presence of more myeloid bodies and absence of the cell connection at 21 days of age compared with the control group (Figure 4A, 4B). No ultrastructural lesions were seen in the AFB_1_+Se group and +Se group when compared with those of the control group (Figure 4C, 4D).

### The number of IgA+ cells in the cecal tonsil

**Figure 5:**
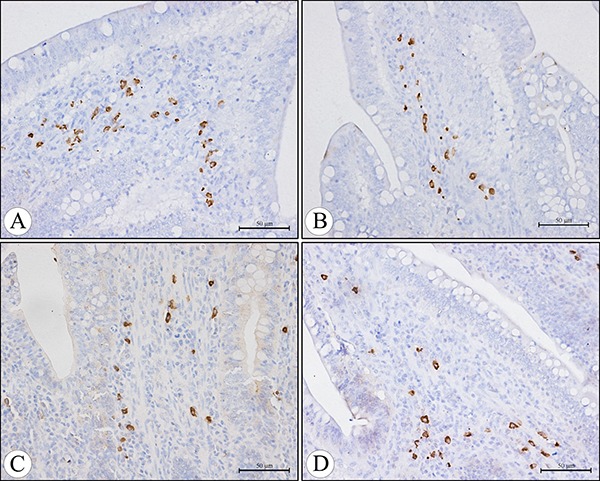
The IgA+ cells in the core of the villi of the cecal tonsil at 21 days of age in the control group (**A**), AFB_1_ group (**B**), AFB_1_+Se group (**C**), and +Se group (**D**) (immunohistochemistry staining). Bars = 50 μm.

**Figure 6:**
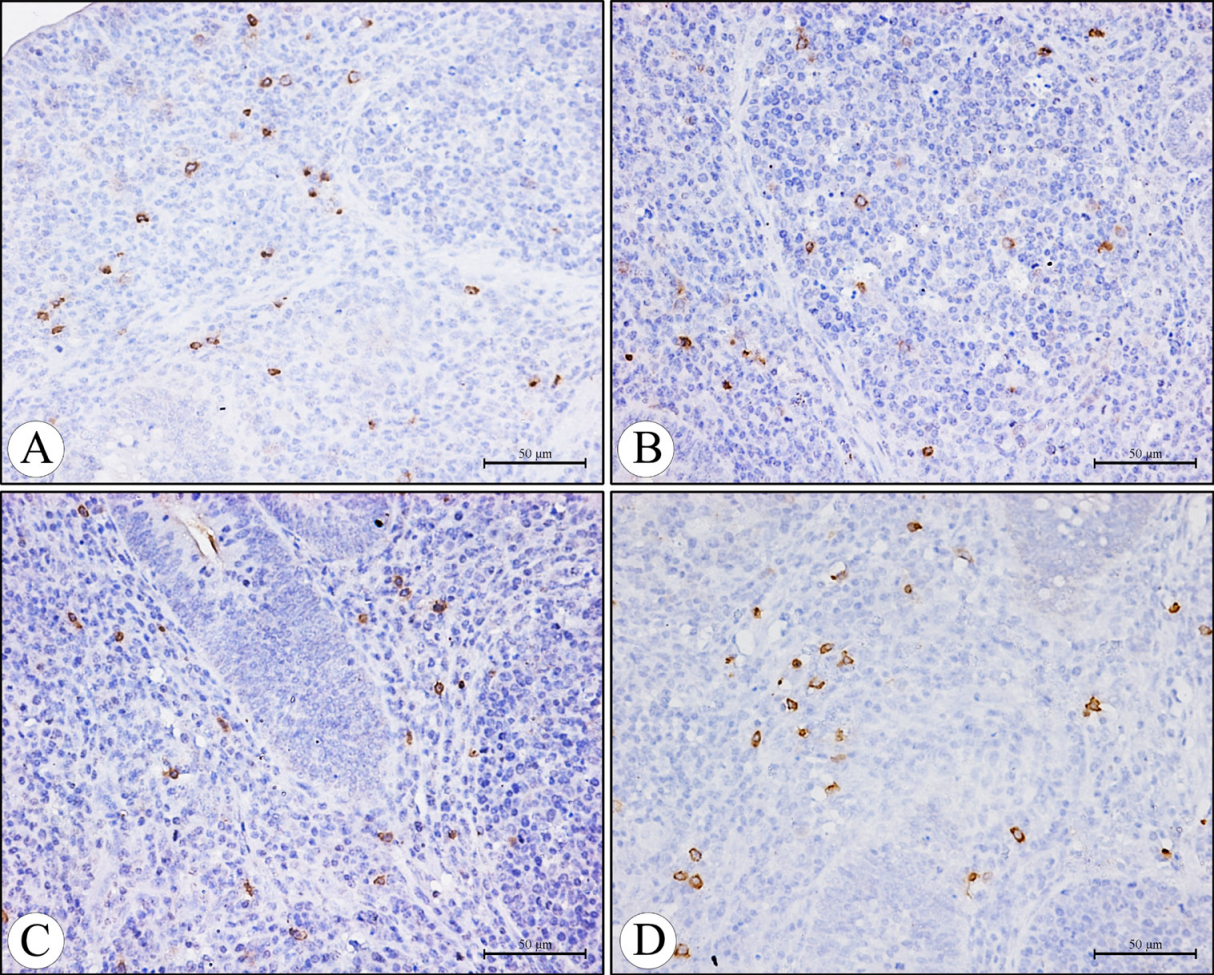
The IgA+ cells in the diffuse lymphoid tissue and lymphatic nodules in the lamina propria of the cecal tonsil at 21 days of age in the control group (**A**), AFB_1_ group (**B**), AFB_1_+Se group (**C**), and +Se group (**D**) (immunohistochemistry staining). Bars = 50 μm.

**Figure 7:**
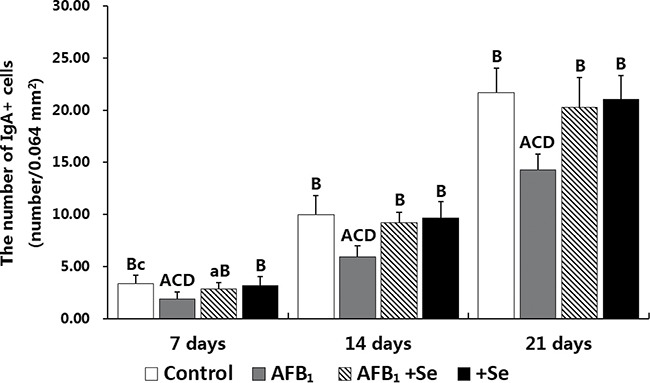
The number of IgA+ cells in the cecal tonsil Note: Data are presented with the means ± standard deviation (*n* = 6). Letters A, B, C and D represent the significant difference (*p* < 0.01) between the group and control group, AFB_1_ group, AFB_1_+Se group, and +Se group, respectively. Letters a, b, c and d represent difference (*p* < 0.05) between the group and control group, AFB_1_ group, AFB_1_+Se group, and +Se group, respectively.

The IgA+ cells were mainly scattered in the core of the intestinal villi and in the diffuse lymphoid tissue and lymphatic nodules of the lamina propria. Exhibiting brown reaction in the cytoplasm, the IgA+ cells were round, oval and irregular in shape, and had scanty cytoplasm and pale stained nuclei (Figures 5, 6). Under light microscope, the decreased number of IgA+ cells were apparently seen in the AFB_1_ group when compared with the control group (Figure 5A, 5B and Figure 6A, 6B). The microscopic quantitative analysis also demonstrated that the numbers of IgA+ cells in the AFB_1_ group were significantly lower (*p <* 0.01) than those in the +Se group and AFB1+Se group from 7 to 21 days of age. Furthermore, compared to the control group, the values in the AFB_1_+Se group showed no significant changes (*p* > 0.05) throughout the experiment except at 7 days of age. In addition, no significant changes in the number of IgA+ cells were observed between the +Se group and control group during the experiment (*p* > 0.05) (Figure 7).

### The expression levels of IgG, IgA, IgM and pIgR mRNA in the cecal tonsil

**Figure 8:**
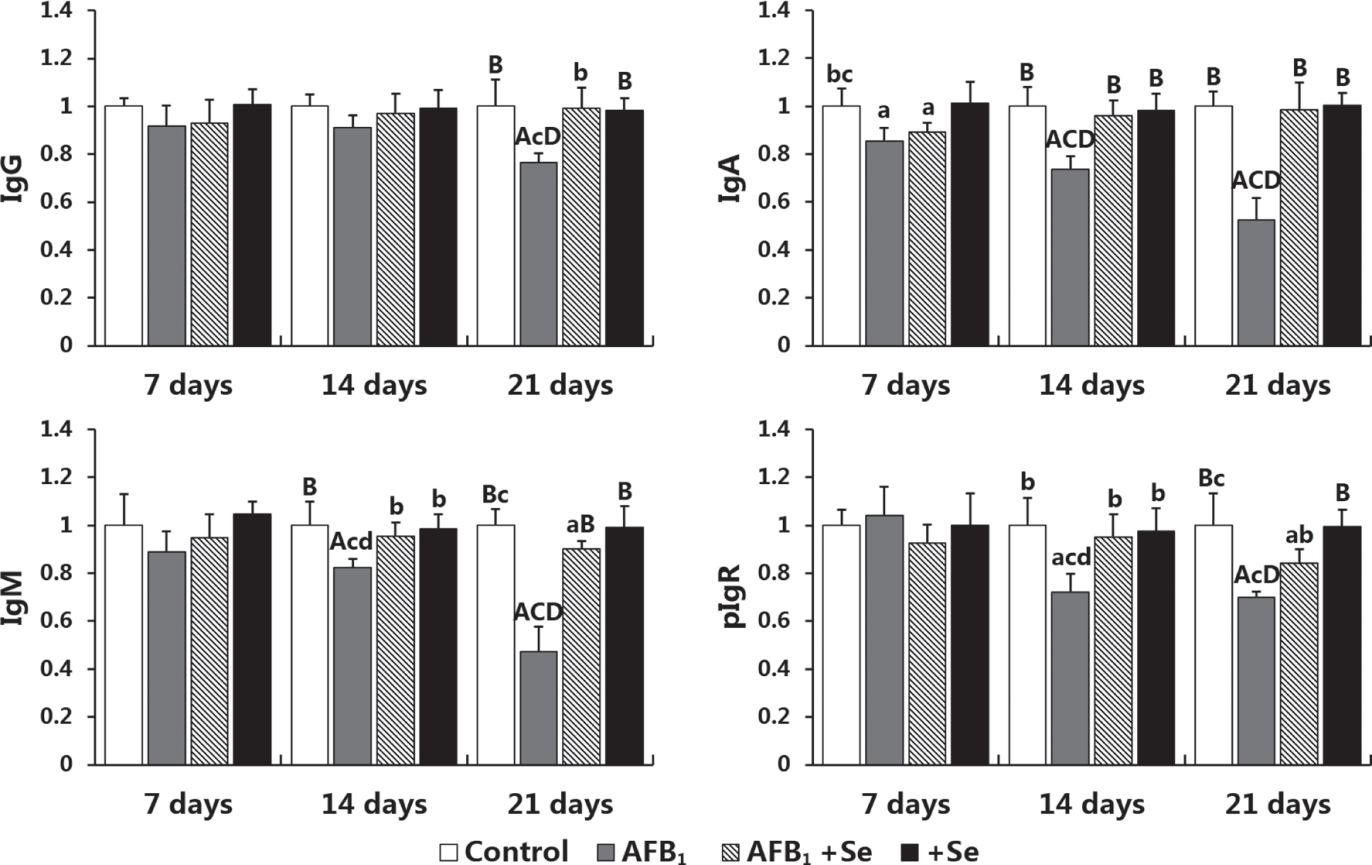
The expression levels of IgG, IgA, IgM and pIgR mRNA in the cecal tonsil Note: data are presented with the means ± standard deviation (*n* = 6). Letters A, B, C and D represent the significant difference (*p* < 0.01) between the group and control group, AFB_1_ group, AFB_1_+Se group, and +Se group, respectively. Letters a, b, c and d represent difference (*p* < 0.05) between the group and control group, AFB_1_ group, AFB_1_+Se group, and +Se group, respectively.

The expression levels of IgG, IgA, IgM and pIgR mRNA are shown in Figure 8. A significant decline in the expression levels of IgG, IgA, IgM and pIgR mRNA in the AFB_1_ group (*p <* 0.01 or *p <* 0.05) at 14 and 21 days of age was noted compared with those in the control group and AFB_1_+Se group except for the IgG mRNA expression at 14 days of age which did not show significant changes (*p >* 0.05). These values in the AFB_1_+Se group showed no significant difference from those in the control group (*p >* 0.05) with the exception of IgA at 7 days, IgM and pIgR at 21 days which were significantly lower than those in the control group (*p <* 0.05). No significant differences in these values were noted between the +Se group and control group during the experiment (*p* > 0.05).

## DISCUSSION

Cecal tonsil, on which nearly half of the lymph nodules are accumulated, is major lymphoid tissue in the avian cecum and is an important component in mucosal immune systems and provides important and unique immune function [24]. Moreover, this organ also has absorptive and digestive functions. In the present research, the depletion of lymphocytes in the lymphatic nodules were demonstrated in the AFB_1_ group. Furthermore, the shedding of microvilli, reduced number of mitochondria cristae in the absorptive cells along with the absence of cell connection were also demonstrated in the AFB_1_ intoxicated broilers. It is well known that the function of cells is closely related to its structure. The lymphocytes in the mucosal lymphatic nodules of digestive tube play a crucial role in mucosal immunity. The functions of microvilli in the absorptive cells are to increase the absorptive areas and to promote digestion and absorption since the glycocalyx on the surface of microvilli contain a large number of enzymes, such as disaccharidase, dipeptidase and transpeptidase [25]. Mitochondria are the structure of energy production in cells, and the inside of each crista of mitochondria is studded with 8.5 nm particles involved in the synthesis of adenosine triphosphate (ATP) [25]. Cell attachment seals off the upper part of the epithelium and this mechanism prevents leakage of material from the lumen into the subepithelial space and vice versa [25]. Therefore, the depletion of lymphocytes in the lymphatic nodules and ultrastructural lesions in the absorptive cells caused by AFB_1_ may eventually impair the normal function of the cecal tonsil. However, it is interesting to find that under light microscope, no shedding of the absorptive cells in the villi of cecal tonsils was observed in the AFB_1_ group, which is consistent with Ledoux's report in the small intestine, when male broilers were exposed to 4 mg /kg AFB_1_ for 3 weeks [26]. However, this result is different from early researches in the jejunum in which the shedding of the absorptive cells occurred on the apical region of villi in the broilers exposure to 0.3 or 0.6 mg/kg AFB_1_ [27, 28]. This discrepancy may be related to the different segments of gastrointestinal tract or AFB_1_ concentration.

Antibodies, made and secreted by B cells, identify extracellular foreign material within the host and help to neutralize and dispose of it. In avian species, they belong to one of three classes: IgA, IgM and IgG (also called IgY) [29]. Being the predominant immunoglobulin isotype in the mucosal tissue, IgA provides mucosal immune protection as a result of its ability to be association with a transmembrane epithelial protein known as pIgR [30]. pIgR has the dual role of transporting locally produced dimeric IgA across mucosal epithelia, and serving as the precursor of secretory component, a glycoprotein that enhances the immune functions of sIgA [31]. sIgA serves as the first line of defense in protecting the intestinal epithelium from enteric toxins and pathogenic microorganisms [32]. Furthermore, IgM antibodies constitute the major component of the natural antibodies and is also the first class of antibodies produced during a primary antibody response [33]. And, IgG predominantly participates in the secondary immune response [34]. In the present study, the significant decrease in the number of IgA+ cells as well as expression levels of IgA, IgG, IgM, and pIgR mRNA at 14 and 21 days of age were observed in the AFB_1_ group, suggesting that 0.6 mg/kg dietary AFB_1_ caused the suppression of the mucosal humoral response in the broiler's cecal tonsil. Similar results were also previously reported in the chicken's small intestine and serum [14, 35]. However, a contrary result revealed that the serum IgA concentration in the AFB_1_-treated ducklings was significantly increased [36]. Thus, the effects of AFB_1_ on antibody production are not conclusive, which might be attributed to the dose, various organs, studied variables, duration of the experiment, and the species of animal/bird.

The cecal tonsil activity depends on the activity of bursa of Fabricius that is the site of B cell development and differentiation in birds [37, 38]. B cells migrate into the lamina propria and mature into IgA-, IgM- and IgG-containing plasma cells [39]. Early researches have demonstrated that AFB_1_ induced the suppression of development, lymphoid depletion, and a decrease of relative weight of bursa of Fabricius [8, 18, 40, 41]. Furthermore, IgA response is highly dependent on T cell function (CD4+ helper T cell) and related cytokines’ (IL-2, IL-4, IL-5, IL-6) help [42–44]. In addition, in the intestinal mucosa, pIgR expression, and hence IgA export depend on transcriptional enhancement by proinflammatory and immunoregulatory cytokines, including IL-1, IL-4, interferon-γ (IFN-γ) and tumor necrosis factor-α (TNF-α) [45, 46]. Recent studies have showed that the number of mature T cell subsets and expression levels of IL-2, IL-4, IL-6, IFN-γ and TNF-α mRNA were decreased in the small intestine and cecal tonsil of broiler induced by AFB_1_ [47, 48]. Finally, Aflatoxin has been found to inhibit RNA polymerase *in vivo* and subsequently to impair protein synthesis, and thereby could inhibit antibody production [49, 50]. Therefore, the decreased number of IgA+ cells and the expression levels of pIgR, IgA, IgG and IgM mRNA observed in the present research might be attributed to the impairment of the bursa of Fabricius, the reduction of T cells, the expression of these cytokines mRNA as well as inhibition of protein synthesis caused by AFB_1_.

Se is an essential trace element for humans and animals. It is generally accepted that this element is a crucial component of several vital metabolic pathways, the antioxidant defense system and the functioning of the immune system [51]. A growing number of evidence revealed that Se has emerged as an important element in the dietary prevention of various toxic agents [52, 53]. Se could inhibit gross and histopathological lesions in most of the organs induced by AFB_1_ [18]. Resent researches suggested that Se exerted its protective effects on the AFB_1_-induced changes in broiler's immune system including the retarded development of spleen, thymus, and bursa of Fabricius [11, 19, 20], reduced percentages of T cell subsets in spleen and thymus [11, 20], decreased contents of serum immunoglobulin [19], lower number of IgA+ cells as well as the decreased contents of sIgA, IgA, IgG, and IgM in the ileum [13]. In the present study, no histological lesions were observed in the AFB_1_+Se group and +Se group. Furthermore, the number of lymphocytes in the lymphatic nodules and IgA+ cells as well as the expression levels of IgA, IgG, IgM, and pIgR mRNA were significantly increased in the AFB_1_+Se group when compared with those in the AFB_1_ group, and no significant differences of these values were observed between the +Se group and control group. These results suggested that 0.4 mg/kg Se supplied with the diet could protect the cecal tonsil from the histological lesion, and the impaired humoral immunity induced by 0.6 mg/kg AFB_1_, in line with early reports in the immune organs, ileum and serum [11, 13, 19, 20]. The exact mechanisms of the protective role of Se on the suppression of humoral immune response of cecal tonsils caused by AFB_1_ needs to elucidate by further research. However, it may be partially associated with following factors: (1) Se could improve the AFB_1_-induced impairment of bursa of Fabricius [19]; (2) Se could increase immunoglobulin synthesis and enhance B-cell function [54, 55]; (3) Se could enhance conjugation of aflatoxins thereby increasing excretion of aflatoxins [56], and repress the formation of AFB_1_-DNA adducts [57]; (4) the protective effect of Se was mediated through a cellular mechanism related to glutathione detoxification pathways [52].

From the present observation it can be concluded that 0.4 mg/kg Se supplied in the diets of broilers could protect the cecal tonsil from the histological lesions, and the suppression of mucosal humoral response induced by 0.6 mg/kg AFB_1_. The present results may be helpful for better understanding of the suppression of AFB_1_-induced mucosal immune response and protective effect of Se against this toxin.

## MATERIALS AND METHODS

### Chickens and diets

One hundred and fifty-six one-day-old healthy avian broilers were purchased from a commercial rearing farm (Wenjiang poultry farm, Sichuan Province, China) and randomly divided into four groups, namely control group, AFB_1_ group (0.6 mg/kg AFB_1_), +Se group (0.4 mg/kg Se supplement), and AFB_1_+Se group (0.6 mg/kg AFB_1_+0.4 mg/kg Se supplement). Our previous studies showed that 0.6 mg/kg AFB_1_ in the diet had obvious adverse effects on broilers, and appropriate dosage of Se (0.4 mg/kg) supplied in the diet could provide optimal protective effects against AFB_1_-induced toxicity in broilers [14, 20, 58]. Based on these researches, toxin concentration (0.6 mg/kg AFB_1_) and supplemented Se level (0.4 mg/kg) were chosen, and sodium selenite was selected as a source of supplemented Se. AFB_1_ was obtained from Pribolab Pte. Ltd. (Singapore, MSS1003). 27 mg AFB_1_ was completely dissolved in 30 mL methanol and then the 30 mL mixture was mixed into the 45 kg corn-soybean basal diet to formulate the AFB_1_ diet of experimental groups containing 0.6 mg/kg AFB_1_. The equivalent methanol was mixed into the corn-soybean basal diet to produce the control diet. Then, the methanol of diets was evaporated at 98 °F (37°C). 1% Feed-grade sodium selenite was mixed into the control diet to formulate +Se and AFB_1_+Se diets containing 0.4 mg/kg Se supplement by a stepwise dilution method. The content of Se (0.332 mg/kg) in the control diet was analyzed by hydride-generation atomic absorption spectroscopy. After preparing the diet, the diets of four groups were analyzed by HPLC (Waters, Milford, MA, USA) and fluorescence detector (Waters Model 2475, Milford) method to ensure the AFB_1_ concentration in the diets. AFB_1_ content was 0.601 mg/kg in the contaminated diet and less than 0.001 mg/kg in the control diet and +Se group. Broilers were provided with drinking water as well as the aforementioned diets *ad libitum* for 21 days. The use of broilers and all experimental procedures involving animals were approved by the Sichuan Agricultural University Animal Care and Use Committee. Nutritional requirements were adequate according to the National Research Council (1994) (National Research Council, 1994) [59] and Chinese Feeding Standard of Chicken (NY/ T33-2004).

### Histological examination by H.E. staining

At the end of 7, 14 and 21 days of experiment, six chickens in each group were euthanized, and cecal tonsils were immediately fixed in 4% paraformaldehyde. After fixation for 24 h, tissues were dehydrated, paraffin embedded, sectioned at 5 μm, and stained with haematoxylin and eosin (H.E) for histological examination. Paraffin sections were also collected to perform immunohistochemistry. The histological structures of the tissues were observed and photographed with a computer supported image system connected to a light microscope (Nikon eclipse 55i, Tokyo, Japan). The number of lymphocytes in the lymphatic nodule was evaluated using Image-Pro Plus 6.0 (Media Cybernetics, Rockville, MD, USA) image analysis software. For each sample, six random fields of 0.0025 mm^2^ in the lymphatic nodules were quantified, respectively. Results were expressed as the average number of lymphocytes in the lymphatic nodule per 0.0025 mm^2^ area.

### Ultrastructral examination by transmission electron microscopy

For the transmission electron microscope (TEM) examination, at the end of 21 days of experiment, the cecal tonsils of three chickens in each group were fixed in 2.5% glutaraldehyde solution for 24 h at 4°C, respectively. The specimens were washed 3 times in PBS (pH 7.4) and fixed in 1% osmium tetroxide for 2 h. After dehydration in a graded series of acetone, they were embedded in Epon 812. Ultrathin sections were cut and stained with uranyl acetate and lead citrate, and examined in a Hitachi H–600 TEM (Japan).

### The IgA+ cells by immunohistochemistry

According to the method described by Liu et al. [39], the paraffin sections of cecal tonsils were dewaxed in xylene, rehydrated through a graded series of ethanol, washed in distilled water and phosphate-buffered saline (PBS), and then blocked for endogenous peroxidase by incubation with 3% H_2_O_2_ in methanol for 15 min. The sections were subjected to antigen retrieval procedure by microwaving in 0.01 M pH 6.0 sodium citrate buffer. Additional washing in PBS was performed before the next 30 min of incubation at 37°C in 10% normal goat serum. The sections were incubated overnight at 4°C with the diluted (1:100) primary antibodies (polyclonal mouse anti-chicken IgA heavy chains) (CAT NO. S8330-01, Southern Biotech, Birmingham, AL, USA). After washing in PBS, the sections were exposed to 1% biotinylated secondary antibody goat antimouse IgG (CAT NO. BA 1050, Boster, Wuhan, China) for 1 h at 37°C. The sections were then incubated with strept avidin-biotin complex (CAT NO. SA 1053, Boster, Wuhan, China) for 30 min at 37°C. To visualize the immunoreaction, the sections were immersed in diaminobenzidine hydrochloride. The sections were lightly counterstained with hematoxylin, dehydrated in ethanol, cleared in xylene and mounted. For the negative controls, representative sections were processed in the same way by replacing primary antibodies by PBS. The stained sections were photographed with a computer supported image system connected to a light microscope (Nikon eclipse 55i, Tokyo, Japan). The number of the IgA+ cells was evaluated using Image-Pro Plus 6.0 (Media Cybernetics, Rockville, MD) image analysis software. For each sample, six random fields of 0.064 mm^2^ were quantified (corresponding approximately to six fields at × 400 magnification), respectively. Results were expressed as the average of IgA+ cells per 0.064 mm^2^ area.

### The expression levels of IgA, pIgR, IgM and IgG mRNA by qRT-PCR

According to the methods described by Wu et al. [24], the expression levels of IgA, pIgR, IgM and IgG mRNA were determined. At 7, 14 and 21 days of the experiment, the cecal tonsils from six chickens in each group were stored in liquid nitrogen, respectively. After adding liquid nitrogen, the cecal tonsils were crushed with a pestle into powder and filled into EP tubes immediately, then stored at −70°C for future usage. Total RNA was extracted from the powder by TriPure isolation reagent (Roche Diagnostics GmbH, Mannheim, Germany). The quality of RNA (A260/A280) was 1.6–2.0 by spectrophotometric analysis and equalized by dilution in RNAase-free water. The mRNA was then reverse transcribed into complementary DNA (cDNA) using Transcription First Strand cDNA Synthesis (Roche Diagnostics GmbH). The cDNA was used as a template for qRT-PCR.

For qRT-PCR reactions, 20 μL mixtures were made by using FastStart Essential DNA Green Master (Roche Diagnostics GmbH) containing 10 μL FastStart Universal SYBR Green Master (ROX), 0.6 μL forward, 0.6 μL reverse primer, 6.8 μL RNAase-free water and 2 μL cDNA. Reaction conditions were set to 10 min at 95°C (first segment, one cycle), 10 s at 95°C and 30 s at T_m_ of a specific primer pair (second segment, 44 cycles) followed by 10 s at 95°C, and 72°C for 10 s (dissociation curve segment) using a Thermal Cycler (Step One Plus, Applied BioSystems, Foster City, CA). Gene expression was analyzed, and β-actin was used as an internal control gene [60, 61]. Sequence of primers was obtained from GenBank of NCBI. Primers were designed with Primer 5 and synthesized by Sangon Biotech (Shanghai, China) (Table 1). The control broilers responses (mRNA amount) were as reference values for between groups comparison within the same control day in each week, respectively. The qRT-PCR data were analyzed with 2^−ΔΔCt^ calculation method of Livak and Schmittgen [62].

**Table 1:** Primer sequences, corresponding accession numbers and sizes of the amplification products

Gene	Primer	Sequences (5′-3′)	Product size (bp)	Accession number
IgA	F	TCGCTGGTATTGATGATGATG	144	S40610
	R	AGTAGTAGGTGGCGGTGTCCT		
pIgR	F	ATGGCTCCGTTAGCATCAAGT	103	AY233381
	R	ACCACAGTCAAGCAACTCCTG		
IgM	F	TCCTTCGTGGACATCTTCATC	161	X01613.1
	R	GTGTAGAGGCCGTTGCTTTG		
IgG	F	CTTAGACGCCAAACTGAGGTG	128	X07174.1
	R	CGTTGAAGTGTTCTTGGAGGA		
β-Actin	F	TGCTGTGTTCCCATCTATCG	150	L08165
	R	TTGGTGACAATACCGTGTTCA		

### Statistical analysis

Statistical analysis was performed with SPSS 16.0 for windows. All parameters determined in this study were presented as mean ± standard deviation (*X*¯ ± SD). Statistical analyses were performed using one-way analysis of variance, and Dunnett T3 was employed for multiple comparisons. A probability value of *p* < 0.05 was considered to be difference, and *p* < 0.01 was considered to be significant difference.
